# Docetaxel induction therapy in locally advanced squamous cell carcinoma of the head and neck

**DOI:** 10.1038/sj.bjc.6600685

**Published:** 2003-01-28

**Authors:** M R Posner, J L Lefebvre

**Affiliations:** 1 Head and Neck Oncology Program, Dana-Farber Cancer Institute, SW430H, 44 Binney street, Boston, MA 02115, USA; 2 Oscar Lambret Center, 3 rue Combemale, 59020 Lille, France

**Keywords:** docetaxel, induction, PF regimens, squamous cell carcinoma of the head and neck

## Abstract

Patients with locally advanced squamous cell carcinoma of the head and neck (SCCHN) are often treated with induction chemotherapy or chemoradiotherapy, but to date without major impact on survival. The combination of cisplatin–5-fluorouracil (5-FU) (PF) has been used as standard induction therapy; however, poor patient survival has stimulated investigation into new agents with potential activity in SCCHN. Docetaxel has significant single-agent activity in SCCHN and has been investigated in combination with PF regimens as induction therapy. The results of six phase II studies of docetaxel–PF regimens (TPF) as induction in locally advanced SCCHN patients are reviewed and reported. Consistently, high 2-year survival rates and overall response rates were demonstrated across the phase II trials in the range 42–82 and 71–100%, respectively. The toxicity profile seen with TPF-based regimens was acceptable. The primary toxicity was neutropenia, which together with gastrointestinal complaints accounted for the majority of adverse events. Given the encouraging phase II experience with TPF-based regimens, two large-scale phase III studies comparing TPF-based regimens with standard PF regimens are underway. The results have significant potential for validating the findings of the phase II studies, demonstrating improved survival and overall response of patients treated with docetaxel-based induction chemotherapy.

Squamous cell cancer of the head and neck (SCCHN) is a significant international problem. Cancer specific to the mouth and pharynx is the sixth most common cancer worldwide, and the third most common cancer among men in developing countries (Johnson, 2001). Men are at higher risk of developing these cancers and are affected 2–3 times as often as women in industrialised countries. There are approximately 400 000 new cases of SCCHN projected annually (Parkin and Muir, 1992). Most SCCHN cases are locally advanced at presentation, with up to 75% of patients having stage III–IV, M0 disease (Dreyfuss *et al*, 1996; Parker, 1996). Stage of disease at diagnosis is regarded as the single most important prognostic factor (Forastiere, 2000). Stage I–II SCCHN is often curable with either surgery or radiotherapy, but it is generally accepted that treatment of locally advanced SCCHN should involve a combined modality approach (Forastiere, 2000).

Surgery and/or radiotherapy remain cornerstones of therapy in patients with locally advanced SCCHN. However, induction chemotherapy and chemoradiotherapy are now firmly integrated in locoregional care. Controlled studies have established cisplatin and continuous-infusion 5-fluorouracil (5-FU) as the standard induction regimen for unresectable locally advanced SCCHN patients (Paccagnella *et al*, 1994; Lefebvre *et al*, 1996; Domenge *et al*, 2000; Pignon *et al*, 2000). Up to 40% of chemonaïve locally advanced SCCHN patients treated with cisplatin–5-FU (PF) have been reported to achieve a complete response, with an overall response rate (ORR) in the range of 85% (Schantz *et al*, 2001). The main toxicities associated with PF-induction therapy are haematological, digestive and mucositis with the majority of events being grade I or II (Paccagnella *et al*, 1994; Domenge *et al*, 2000). Despite inducing locoregional control, high response rates and a significant improvement in survival, PF induction therapy is associated with a relatively poor absolute rate of patient survival, which is similar to that seen with intensive chemoradiotherapy regimens (Paccagnella *et al*, 1994; Lefebvre *et al*, 1996; Domenge *et al*, 2000; Pignon *et al*, 2000). The dose-intensive cisplatin–5-FU–leucovorin (PFL) combination was subsequently developed in an attempt to improve complete response rates to induction chemotherapy and thus survival. The addition of leucovorin resulted in higher complete response rates at the expense of increased toxicity (Vokes *et al*, 1990; Schneider *et al*, 1995; Clark *et al*, 1997).

The need for further improvements in SCCHN care has stimulated intensive investigation into new agents with potential in induction chemotherapy and chemoradiotherapy regimens. New agents such as epidermal growth factor receptor (EGFR) inhibitor ZD1839 (Iressa™), antibody C225 (Cetuximab™) and inhibitor OSI-774 (Tarceva™), in addition to farnesyl transferase inhibitors, have shown some promise in early studies. Although they are relatively ineffective as single agents, their combination with other agents (radiation and/or cytotoxic drugs) is under evaluation and may be of interest. UFT (a mixed compound of 5-FU and uracil) has shown promising single-agent activity, but combination therapy has yet to be studied. Alpha interferon has been tested as a potentiator of PFL or PF chemotherapy, but a phase III trial has failed to demonstrate any additional advantages from this approach (Vokes *et al*, 1995; Schrijvers *et al*, 1998). Perhaps the most promising agents have been the taxanes. In particular, the taxane docetaxel has demonstrated significant single-agent activity in recurrent SCCHN (Catimel *et al*, 1994; Dreyfuss *et al*, 1996; Couteau *et al*, 1999). Docetaxel is similar to paclitaxel with respect to its general mechanism of action (tubulin stabilization and cell cycle arrest), and the two share a number of pharmacological characteristics (Colevas and Posner, 2001). However, docetaxel and paclitaxel have somewhat different pharmacodynamics and toxicities that may be important in combination therapy programmes. Docetaxel, for example, has a greater affinity for tubulin, a longer intracellular half-life, and promotes microtubule stabilization at lower drug concentrations (Lavelle *et al*, 1995). The major toxicity associated with docetaxel is highly predictable myelotoxicity (Posner, 2001a). More importantly, neuropathy, a dominant side effect of cisplatin, is minimal with docetaxel-containing regimens, but can be significant in paclitaxel-containing regimens (Schrijvers and Vermorken, 2000; Colevas and Posner, 2001; Posner, 2001a). The differing mechanisms of action, and relative nonoverlapping toxicities compared with PF, have prompted interest in the addition of docetaxel to PF regimens.

## potential of docetaxel in SCCHN

In early preclinical studies, docetaxel demonstrated pronounced *in vitro* and *in vivo* cytotoxicity against a variety of human cancer cell lines, particularly head and neck cancer (Braakhuis *et al*, 1994). In fact, docetaxel was more effective than cisplatin in inhibiting the growth of two SCCHN xenograft models (HNX-14C and HNX-22B) (Braakhuis *et al*, 1994). Further preclinical evidence for docetaxel efficacy has been derived from studies employing a murine model, representative of human head and neck cancer (Sommer *et al*, 2001). From the drugs investigated (cisplatin, carboplatin, docetaxel, methotrexate, 5-FU), as single agents or in combination, an ifosfamide–docetaxel combination produced the best tumour-free survival.

The clinical efficacy and safety of docetaxel in SCCHN was initially established in patients with metastatic or recurrent/incurable SCCHN. In three phase II studies ( Table 1

**Table 1:** Phase II studies with single-agent docetaxel in patients with SCCHN

**Study reference**	**Dose/schedule**	**N (evaluable patients)**	**Response rate (%)**
Catimel *et al* (1994)	100 mg m^−2^	37	32
	d1 q3w		
Dreyfuss *et al* (1996)	100 mg m^−2^	29	42
	d1 q3w		
Couteau *et al* (1999)	100 mg m^−2^	21	21
	d1 q3w		

), single-agent docetaxel (100 mg m^−2^) administered on day 1 every 3 weeks produced response rates ranging from 21 to 42% (Catimel *et al*, 1994; Dreyfuss *et al*, 1996; Couteau *et al*, 1999). As expected, grade III–IV neutropenia was the principal toxicity in these studies. These results compare favourably with other single agents in the management of SCCHN (Schoffski *et al*, 1998; Posner, 2001a).

Phase I/II studies combining docetaxel with cisplatin and/or 5-FU were undertaken in recurrent or metastatic SCCHN patients to look for improved efficacy and safety. In two trials, docetaxel–5-FU resulted in ORRs of 24 and 27% (Colevas *et al*, 2000; Tubiana-Mathieu *et al*, 2000). Docetaxel–cisplatin appeared to be a more effective combination, with overall response rates ranging from 33 to 76% (Kienzer *et al*, 1998; Schoffski *et al*, 1999; Specht *et al*, 2000; Glisson *et al*, 2002). In the trial conducted by the European Organisation for the Research and Treatment of Cancer (EORTC), docetaxel (100 mg m^−2^) plus cisplatin (75 mg m^−2^) produced a response rate of 54% (Schoffski *et al*, 1999). Of the 44 patients enrolled in this trial, 22 were chemotherapy-naïve and the response rate in this group was 86%. As with single-agent docetaxel, the main toxicity in each of the combination studies was myelosuppression (Kienzer *et al*, 1998; Schoffski *et al*, 1999; Colevas *et al*, 2000; Specht *et al*, 2000; Tubiana-Mathieu *et al*, 2000; Glisson *et al*, 2002). Mucositis was more commonly seen in the combination studies (particularly docetaxel–5-FU) than with single-agent docetaxel.

## Docetaxel induction therapy in SCCHN: phase II results

The promising response rates and tolerability profiles exhibited in recurrent or metastatic SCCHN patients treated with docetaxel–cisplatin led to the investigation of docetaxel regimens as induction therapy for locally advanced disease. Two phase II induction studies with docetaxel 75 mg m^−2^ plus cisplatin 75 or 100 mg m^−2^ i.v. every 3 weeks followed by definitive radiotherapy in patients with locally advanced SCCHN have been reported (Mel *et al*, 2000; Caponigro *et al*, 2001). In an intention-to-treat analysis, objective responses of 62% and 46% were seen in patients during the induction phase, with corresponding complete response rates of 17% (Mel *et al*, 2000) and 11% (Caponigro *et al*, 2001), respectively. Grade III–IV neutropenia was experienced by 74% of patients in one study, although febrile neutropenia affected only 17% of patients (Mel *et al*, 2000). In the second study, 61% of patients experienced grade III–IV neutropenia, which was complicated by fatal sepsis in 2 (4%) patients (Caponigro *et al*, 2001). The authors suggest that these toxic deaths might have been related to the higher dose of cisplatin (100 mg m^−2^) used in their study (Caponigro *et al*, 2001). The incidences of grade III–IV nonhaematological toxicity, including gastrointestinal complications, were relatively low in both studies. These results, in a heterogeneous group of patients with moderate-to-poor performance status, were similar to the data seen with PF. With data from recurrent patients, these studies demonstrate that the docetaxel–cisplatin combination has interactive efficacy similar to that seen with PF.

Rather than substituting cisplatin or 5-FU with a newer SCCHN-active agent such as docetaxel, in the curative setting, most research is now focused on the addition of docetaxel to PF-based regimens. Studies involving docetaxel–PF-based regimens demonstrate a high likelihood of superior clinical benefit *vs* current treatment options in terms of response rate and survival.

### Docetaxel plus modified PFL regimens

Three trials conducted at the Dana-Farber Cancer Institute administered docetaxel with PFL in SCCHN patients with advanced disease, who were potentially curable ( Table 2

**Table 2:** Docetaxel plus modified PFL induction regimens for locally advanced SCCHN

**Study reference**	**Number of patients entered**	**Patient/disease characteristics**	**Regimen**	**OR^a^ (%)**	**CR^a^ (%)**	**Grade III–IV toxicities in ⩾5% of patients or cycles (%)**
Colevas *et al*, (1998) Phase I/II (*n*=24)	23	Previously untreated stage III or nonmetastatic stage IV SCCHNECOG PS ⩽2	TPFL-5: docetaxel 25–60 mg m^−2^ i.v. d1; cisplatin 25 mg m^−2^ day^−1^ CIVI d1–5; 5-FU 700 or 800 mg m^−2^ day^−1^ CIVI d2–5; leucovorin 500 mg m^−2^ day^−1^ CIVI d1–5 Repeat q4wk for up to three cycles, followed by definitive BID RT	100	61	% of cycles at the MTD of docetaxel 60 mg m^−2^ i.v. (*n*=45): mucositis (46); neutropenia (22); febrile neutropenia (22); nausea (11); diarrhoea (11); infection (7); renal tubular (7)
Colevas *et al* (1999) Phase II (*n*=30)	30	Previously untreated stage III or nonmetastatic stage IV SCCHNECOG PS ⩽2	TPFL-4: docetaxel 60 mg m^−2^ i.v. d1; cisplatin 31.25 mg m^−2^ day^−1^ CIVI d1–4; 5-FU 700 mg m^−2^ day^−1^ CIVI d1–4; leucovorin 100 mg oral LD×2 (6 and 12 h before docetaxel) then 500 mg m^−2^ day^−1^ CIVI day 1–4 Repeat q4wk for up to three cycles, followed by definitive BID RT	93	63	% of TPFL-4 cycles: mucositis (48); nausea/vomiting (15); neutropenia (8); thrombocytopenia (6); anorexia (6); diarrhoea (5); infection (5); renal toxicity (5)
Colevas *et al* (2002) Phase I/II (*n*=34)	34	Previously untreated stage III or nonmetastatic stage IV SCCHN ECOG PS 0 or 1	Outpatient TPFL: docetaxel 60–95 mg m^−2^ day^−1^ i.v. d1; cisplatin 100 mg m^−2^ day^−1^ i.v. d1; 5-FU 700 mg m^−2^ d^−1^ CIVI d1–4; leucovorin 100 mg oral LD×2 then 500 mg m^−2^ day^−1^ CIVI d1–4 Repeat q3wk for up to three cycles in responders, followed by definitive BID RT	94	44	% of patients at the MTD of docetaxel 90 mg m^−2^ i.v. (*n*=15): neutropenia (67); mucositis (33); nausea (13); anorexia (13); fatigue (13); febrile neutropenia (7); thrombocytopenia (7); diarrhoea (7); hepatotoxicity (7); hyponatraemia (7); pain (7)

Abbreviations: SCCHN=squamous cell carcinoma of the head and neck; OR=overall response; CR=complete response; ECOG=Eastern Cooperative Oncology Group; PS=performance status; TPFL=docetaxel/cisplatin/5-fluorouracil/leucovorin; i.v.=intravenously; 5-FU=5-fluorouracil; CIVI=continuous intravenous infusion; LD=loading dose; RT=radiotherapy; MTD=maximum tolerated dose; BID=Twice Daily Hyperfractionated

a For the evaluable population.

) (Colevas *et al*, 1998,^1999^,^2002^). In the first study, TPFL-5, 23 patients with stage III–IV SCCHN received docetaxel 25–60 mg m^−2^ d1 (day 1), cisplatin 25 mg m^−2^ d1–5, 5-FU 700 mg m^−2^ (*N*=21) or 800 mg m^−2^ (*N*=2) d2–5 and leucovorin 500 mg m^−2^ d1–5 every 4 weeks for three cycles (Colevas *et al*, 1998). The ORR prior to definitive, twice daily radiation therapy was 100%, consisting of 14 complete responses (CRs; 61%) and nine partial responses (PRs; 39%). Updated survival data (median follow-up 43 months) revealed both the 3-year overall survival and disease-free survival rates to be 78% (Posner *et al*, 2000). Approximately 40% of patients receiving the maximum tolerated dose (MTD) of docetaxel were hospitalised for neutropenia despite administration of granulocyte colony-stimulating factor (G-CSF) d5–10 and ciprofloxacin d5–15 (Colevas *et al*, 1998). Mucositis was the most prominent grade III–IV nonhaematological toxicity (46% of cycles), followed by nausea (11% of cycles) and diarrhoea (11% of cycles). In all, 14 patients (61%) developed mild neuropathy 1–8 months after the first dose of chemotherapy.

In an attempt to reduce hospitalisation, the TPFL-4 regimen was developed in which G-CSF was started earlier and treatment was compressed into the first 4 days of the 4-week cycle (Colevas *et al*, 1999). In all, 30 stage III–IV SCCHN patients received two loading doses of oral leucovorin 100 mg followed by docetaxel (60 mg m^−2^) d1 and then infusions of cisplatin (31.25 mg m^−2^ day^−1^), 5-FU (700 mg m^−2^ day^−1^) and leucovorin (500 mg m^−2^ day^−1^) over 4 days. G-CSF was given d4–10 and ciprofloxacin on d5–15. The ORR was 93%, with 63% CRs and 30% PRs. Primary tumour site clinical and pathological responses were 93 and 68%, respectively. The originally reported 2-year overall survival and disease-free survival rates were 87 and 57%, respectively (Colevas *et al*, 1999). A more recent analysis (median follow-up 30 months) reported the 2-year overall survival and disease-free survival rates as 83 and 53%, respectively (Posner *et al*, 2000). Haematological toxicity was generally mild, with grade III–IV neutropenia, thrombocytopenia, and anaemia reported during 8, 6 and 2% of cycles, respectively (Colevas *et al*, 1999). Mucositis was the most prominent nonhaematological grade III–IV event (48% of cycles), followed by nausea/vomiting (15% of cycles). These values represent major reductions in haematological and infectious toxicities compared with patients in TPFL-5. As a result, hospitalization was reduced to approximately 14%, the main cause of which was dehydration.

The op-TPFL trial, an outpatient study, represents a further docetaxel–TPFL regimen modification (Colevas *et al*, 2002). The objective of the study was to allow stage III–IV SCCHN patients to receive TPFL chemotherapy at home with intensive nursing support. Two doses of leucovorin 100 mg were given as oral loading. This was followed by docetaxel 60–95 mg m^−2^ d1, cisplatin 100 mg m^−2^ d1, 5-FU 700 mg m^−2^ d1–4 and leucovorin 500 mg m^−2^ day 1–4 in a 3-week cycle. G-CSF and antibiotics were administered, starting 6 h after the end of chemotherapy. The MTD of docetaxel was 90 mg m^−2^ with G-CSF support. The ORR was 94% and the CR 44% in the 34 treated patients. At a median follow-up of 12 months, 27 (79%) patients were alive, with 18 (53%) patients free of disease progression. Neutropenia and mucositis were the most frequently observed toxicities. Of the 15 patient who received 42 cycles administered at the MTD, grade III–IV neutropenia and mucositis were observed in 15 (67%) and 5 (33%) patients, respectively. Febrile neutropenia occurred in only 1 (7%) patient. These results suggest that home-administered op-TPFL is a viable clinical option.

### Docetaxel plus PF regimens

The TPFL regimens are intensive and associated with considerable toxicity. Patients routinely require G-CSF support and a significant proportion are hospitalised either for treatment or for treatment-related toxicity. In many patients age and comorbidities rule out such dose-intensive chemotherapy regimens. Intermediate dose-intensive regimens involving docetaxel in combination with PF may be equally efficacious, providing alternatives in these patient groups. Three trials in which this intermediate-dose approach was investigated are discussed here and summarised in Table 3

**Table 3:** Docetaxel plus PF induction regimens for locally advanced SCCHN

**Study**	**Number of patients entered**	**Patient/disease characteristics**	**Regimen**	**OR^a^ (%)**	**CR^a^ (%)**	**Grade III–IV toxicities in ⩾5% of patients (%)**
TAX 708 Posner *et al* (2001b) Phase I/II (*n*=43)	43	Locally advanced SCCHN ECOG PS 0 or 1 CT-naïve and no prior RT or surgery for SCCHN	Docetaxel 75 mg m^−2^ i.v. d1; Cisplatin 75 mg m^−2^ or 100 mg m^−2^ i.v. d1; 5-FU 1000 mg m^−2^ day^−1^ CIVI d1–4 Repeat q3wk for up to three cycles, followed by institution-specific definitive therapy	93	40	Neutropenia (95); stomatitis (30); hypomagnesaemia/hypocalcaemia (30); febrile neutropenia (19); nausea (19); diarrhoea (9); vomiting (7); dehydration (7); thrombocytopenia (5); neurological-hearing (5); liver enzyme abnormalities (5)
						
Janinis *et al* (2001) Phase II (*n*=20)	20	Locally advanced SCCHN WHO PS 0–2 CT- and RT-naïve	Docetaxel 80 mg m^−2^ i.v. d1; Cisplatin 40 mg m^−2^ i.v. D2,3; 5-FU 1000 mg m^−2^ day^−1^ CIVI d1–3 Repeat q4wk for up to four cycles, followed by definitive RT	90	20	Leucopenia (25); febrile neutropenia (10); Infection (10); diarrhoea (5)
						
TAX 017HN Schrijvers *et al* (1999) Phase I/II (*n*=48)	48^b^	Locally advanced SCCHN ECOG PS 0 or 1 No prior cancer treatment	Docetaxel 75 mg m^−2^ i.v. d1; Cisplatin 75 mg m^−2^ (level I) or 100 mg m^−2^ (level II) i.v. d1; 5-FU 750 mg m^−2^ day^−1^ CIVI d1–5 Repeat q3wk for up to four cycles, followed by definitive RT	71	0	Level I (*n*=17): neutropenia (76); infection (13); stomatitis (6); diarrhoea (6) Level II (*n*=11): neutropenia (63); infection (18); stomatitis (9); nausea (9); vomiting (9)

Abbreviations: SCCHN=squamous cell carcinoma of the head and neck; OR=overall response; CR=complete response; ECOG=Eastern Cooperative Oncology Group; PS=performance status; CT=chemotherapy; RT=radiotherapy; i.v.=intravenously; 5-FU=5-fluorouracil; CIVI=continuous intravenous infusion; WHO=World Health Organization; NR=not reported.

a For the intention-to-treat population

b Unpublished data.

.

In the TAX 708 study, 43 chemonaïve patients with locally advanced SCCHN received docetaxel 75 mg m^−2^ d1, cisplatin 75 mg m^−2^ (level I; *N*=13) or 100 mg m^−2^ (level II; *N*=30) d1 and continuous 5-FU 1000 mg m^−2^ day^−1^ d1–4 (Posner *et al*, 2001b). Ciprofloxacin was administered to all patients day 5–15 and G-CSF was permitted to manage febrile neutropenia or treatment-delaying myelosuppression. The ORR was 93% and the CR 40%. A complete clinical response was seen in 57% of assessable tumours (Posner *et al*, 2001b). At a recent follow-up (median 26 months), the 2-year overall survival rate was 82% (Posner *et al*, 2001c). Of the grade III–IV toxicities, neutropenia affected 95% of patients; however, febrile neutropenia and infection affected only 19% and 2% of patients, respectively (Posner *et al*, 2001b). Other grade III–IV toxicities include stomatitis (30%), transient renal problems (30%), nausea (19%) and diarrhoea (9%) (Posner *et al*, 2001b).

In a similar study (TAX 017HN), 48 patients with locally advanced SCCHN received docetaxel 75 mg m^−2^ and cisplatin 75 mg m^−2^ (level I) or 100 mg m^−2^ (level II) d1, followed by continuous infusions of 5-FU 750 mg m^−2^ day^−1^ over 5 days (Schrijvers *et al*, 1999). Owing to infectious complications seen in the first 18 patients, ciprofloxacin was added from d5 to 15. Prior to this, six patients developed infections leading to hospitalisation. Grade III–IV neutropenia was reported in 88 and 63% of patients in the level I and II groups, respectively. An ORR of 71% was observed. At a recent follow-up (median 24 months), the 2-year overall survival rate was 42% (Posner *et al*, 2001c).

Janinis *et al* (2001) administered docetaxel 80 mg m^−2^ d1, cisplatin 40 mg m^−2^ d2 and d3 and continuous 5-FU 1000 mg m^−2^ day^−1^ d1–3 every 4 weeks in chemonaïve patients with locally advanced SCCHN. All patients received G-CSF d4–9. A maximum of four chemotherapy cycles were allowed and radiation therapy was planned after completion of chemotherapy. The ORR postchemotherapy was 90% with a CR of 20%. After radiotherapy, the ORR was 95% with a CR that increased to 73%. After a median follow-up of 36 months, median disease-free and overall survival had not been reached. The 2-year survival rate was 60%. Grade III–IV toxicity was limited to leucopenia (25%), febrile neutropenia (10%), grade IV infection (10%) and grade IV diarrhoea (5%). The most common acute nonhaematological toxicities included alopecia, mucositis and peripheral sensory neuropathy. Alopecia was reversible, and mucositis was mild and did not require hospitalisation. Peripheral neuropathy was seen in 25% of patients, but was of mild degree with late occurrence.

## Ongoing phase III induction studies incorporating docetaxel

It is apparent that docetaxel, when used in conjunction with standard induction therapy (cisplatin, 5-FU with or without leucovorin), has significant activity in locally advanced SCCHN. Consistently high 2-year survival rates and ORRs have been observed across phase II studies in the range 42–82 and 71–100%, respectively (Posner *et al*, 2001c). Each of the phase II studies discussed has used a slightly different regimen with varying concentrations of chemotherapy in 3- or 4-week cycles. The study results have suggested somewhat different response rates, overall survival rates and toxicity profiles. This may be attributed to factors such as differences in patient selection or subtle differences in schedule-related multidrug interactions.

Given the encouraging phase II experience with docetaxel induction regimens, and the need to further investigate the various regimens in a randomised, controlled setting, phase III studies of two TPF-based regimens *vs* PF are warranted and are under way (Posner *et al*, 2000). A phase III trial (TAX 324) is being conducted mainly in the USA, with some centres in Europe and South America. This trial is evaluating three cycles of TPF (docetaxel 75 mg m^−2^ d1, cisplatin 100 mg m^−2^ d1 and 5-FU 1000 mg m^−2^ d1–4 every 3 weeks) *vs* three cycles of PF (cisplatin 100 mg m^−2^ d1 and 5-FU 1000 mg m^−2^ d1–5 every 3 weeks). This trial comprises a sequential therapy design incorporating postinduction chemoradiotherapy. Patients in both arms are treated with definitive chemoradiation, with weekly carboplatin for a maximum of seven doses following induction chemotherapy. A second study conducted by the EORTC (TAX 323) is evaluating four cycles of TPF (docetaxel 75 mg m^−2^ d1, cisplatin 75 mg m^−2^ d1 and 5-FU 750 mg m^−2^ d1–5 every 3 weeks) *vs* four cycles of PF (cisplatin 100 mg m^−2^ d1 and 5-FU 1000 mg m^−2^ d1–5 every 3 weeks). A 7-week course of radiotherapy is to commence within 4–7 weeks of completing the last chemotherapy cycle. These ongoing, large-scale, randomised trials are expected to confirm the positive benefits of adding docetaxel to PF-based regimens, as already demonstrated in the phase II setting.

## Docetaxel-based chemoradiation regimens for locally advanced disease

In recent controlled studies, PF-based chemoradiation regimens, used as induction or definitive therapy, have demonstrated improved efficacy compared with conventional or hyperfractionated radiotherapy alone in locally advanced SCCHN (Vokes *et al*, 1995; Brizel *et al*, 1998). The activity of docetaxel in SCCHN, coupled with the drug's *in vitro* radiosensitizing properties (Creane *et al*, 1999; Mason *et al*, 1999; Pradier *et al*, 2001), provide the basis for clinical trials with docetaxel as a component of concurrent or alternating chemoradiation in locally advanced SCCHN (Koukourakis *et al*, 1999; Hesse *et al*, 2000; Tishler *et al*, 2002).

Docetaxel-based chemoradiation regimens have been shown to be feasible and active as induction and adjuvant therapy. The phase I/II study by Tishler *et al* (2002) considered daily radiation concurrent with weekly docetaxel in 21 patients with stage III–IV SCCHN, who responded poorly to induction chemotherapy, and demonstrated an ORR of 86% (CR 57%). Radiation was delivered at 2 Gy day^−1^, to a total dose of 66–74 Gy. The MTD of weekly docetaxel in this regimen was 25 mg m^−2^. Mucositis was the major acute toxicity, occurring at grade III in all patients receiving docetaxel 25 mg m^−2^. Swallowing problems represented the main long-term toxicity, although no patient remained feeding-tube dependent. Three-year survival was >60% in this poor prognosis group of patients.

Chemoradiation therapies with docetaxel–cisplatin have achieved an ORR as high as 100% (Varveris *et al*, 1999; Budach *et al*, 2000). The chemoradiation protocol considered by Budach *et al* (2000) was devised to reduce oral toxicity. A total of 15 patients with inoperable, recurrent head and neck cancer received three cycles of docetaxel (50–60 mg m^−2^ d1) and cisplatin (15 mg m^−2^ d2–5) during weeks 1, 4 and 7 alternating with two courses of radiotherapy (5×2 Gy; total dose 40 Gy) in weeks 2–3 and 5–6. The recommended dose of docetaxel was 50 mg m^−2^, as 60 mg m^−2^ led to unacceptable systemic toxicity in the first 12 patients treated. An ORR of 92% was achieved, with acceptable levels of oral toxicity.

Varveris *et al* (1999) demonstrated that the radiosensitizing effect of docetaxel–cisplatin on hyperfractionated radiotherapy enables an ORR of 100% (CR 59%). Docetaxel and cisplatin were both given at 30 mg m^−2^ twice weekly to 54 patients. This dose was reduced to 15–20 mg m^−2^ because of severe acute toxicity in the first 10 patients. Major toxicities included grade III–IV mucositis (86%), grade I–III hypersensitivity reactions (11%) and grade III myelotoxicity (11%).

Further studies are needed to elucidate the relative efficacy and toxicity of these concurrent radiation regimens *vs* the traditional sequential approach of induction chemotherapy followed by definitive radiotherapy.

## Conclusions

Unmet needs in SCCHN care have stimulated intensive investigation into new agents with potential in induction chemotherapy and chemoradiotherapy regimens. Docetaxel has emerged as one of the most active agents in SCCHN, and is particularly efficacious as a component of induction therapy in locally advanced disease. The results of phase II studies investigating TPF induction regimens demonstrate consistently high 2-year survival rates and ORRs across all trials, in the range of 42–82 and 71–100%, respectively (Posner *et al*, 2001c). An acceptable toxicity profile is seen with TPF-based regimens, with neutropenia and gastrointestinal complaints accounting for the majority of adverse events. The encouraging phase II results are being validated in the ongoing, large-scale, randomised phase III studies comparing TPF with PF regimens.
